# Long COVID: neurological manifestations - an updated narrative review

**DOI:** 10.1590/1980-5764-DN-2023-0076

**Published:** 2024-02-09

**Authors:** José Wagner Leonel, Gabriella Cunha Vieira Ciurleo, Alissa Moura Formiga, Thais de Maria Frota Vasconcelos, Marcello Holanda de Andrade, Werbety Lucas Queiroz Feitosa, Antônio Alves Sobreira-Neto, Chiara Gübel Portugal, Lorenzo Marinho Morais, Samuel Cavalcante Marinho, Emanuel de Assis Bertulino Martins Gomes, Esther de Alencar Araripe Falcão Feitosa, Emmanuelle Silva Tavares Sobreira, Reinaldo Barreto Oriá, Manoel Alves Sobreira-Neto, Pedro Braga-Neto

**Affiliations:** 1 Universidade Federal do Ceará, Fortaleza CE, Brazil.

**Keywords:** Post-Acute COVID-19 Syndrome, Cognitive Dysfunction, Cognition, Síndrome Pós-COVID-19 Aguda, Disfunção Cognitiva, Cognição

## Abstract

Infection with the SARS-CoV-2 virus can lead to neurological symptoms in the acute phase and in the Long COVID phase. These symptoms usually involve cognition, sleep, smell disorders, psychiatric manifestations, headache and others. This condition is more commonly described in young adults and women. This symptomatology can follow severe or mild cases of the disease. The importance of this issue resides in the high prevalence of neurological symptoms in the Long COVID phase, which entails significant morbidity in this population. In addition, such a condition is associated with high health care costs, with some estimates hovering around 3.7 trillion US dollars. In this review, we will sequentially describe the current knowledge about the most prevalent neurological symptoms in Long COVID, as well as their pathophysiology and possible biomarkers.

## RESUMO.

A infecção pelo vírus SARS-CoV-2 pode levar a sintomas neurológicos na fase aguda e na fase de COVID longa. Esses sintomas geralmente envolvem cognição, sono, distúrbios do olfato, manifestações psiquiátricas, dor de cabeça e outros. Esta condição é mais comumente descrita em adultos jovens e mulheres. A sintomatologia pode acompanhar casos graves ou leves da doença. A importância desta questão reside na elevada prevalência de sintomas neurológicos na fase de COVID longa, o que acarreta morbilidade significativa nesta população. Além disso, tal condição está associada a elevados custos de cuidados de saúde, com algumas estimativas em torno de 3,7 trilhões de dólares americanos. Nesta revisão, descrevemos sequencialmente o conhecimento atual sobre os sintomas neurológicos mais prevalentes na COVID longa, bem como sua fisiopatologia e possíveis biomarcadores.

## INTRODUCTION

Infection with the SARS-CoV-2 virus can lead to neurological symptoms in the acute phase and in the long COVID phase^1^ ^,^ ^2^. These symptoms usually involve cognition, sleep, smell disorders, psychiatric manifestations, headache and others^3^ ^,^ ^4^ ^,^ ^5^. This condition is more commonly described in young adults and women^6^. The duration of these symptoms is unknown in most studies, while other authors refer to the possibility of improvement after one year of infection^7^. The symptomatology can vary from severe to mild cases of the disease^8^. A systematic review and meta-analysis of 81 studies evaluating patients 12 weeks or more after a clinical diagnosis of COVID-19 reported that the most frequent symptoms of long COVID were fatigue and cognitive impairment, which was associated with considerable functional impairment^9^. The importance of this issue resides in the high prevalence of neurological symptoms in the long COVID phase, which entails significant morbidity in this population^10^. In addition, such a condition is associated with high health care costs, with some estimates hovering around 3.7 trillion US dollars^11^. In this review, we will sequentially describe the current knowledge about the most prevalent neurological symptoms in long COVID, as well as their pathophysiology and possible biomarkers.

## COGNITIVE SYMPTOMS

Cognitive impairment is common in long COVID and can occur after mild or severe cases of COVID-19^7^ ^,^ ^12^ ^,^ ^13^ ^,^ ^14^ ^,^ ^15^. These symptoms normally involve attention, memory, executive functions and processing speed complaints^3^ ^,^ ^8^ ^,^ ^13^ ^,^ ^14^ ^,^ ^15^ ^,^ ^16^. Cognitive complaints and cognitive impairment associated with COVID-19 have been described in the different phases of COVID-19^17^. A Chinese study, for example, assessed the cognition of 29 patients with COVID-19 through questionnaires that were self-completed by patients remotely, and they correlated cognitive complaints with elevated levels of C-reactive protein (PCR) during the acute phase of the disease^1^. Another study used positron emission tomography and demonstrated that, in the acute phase of the disease, encephalopathy was associated with severe conditions and akinetic mutism was associated with frontal hypometabolism in the brain^18^. Jaywant et al. evaluated 57 patients hospitalized with COVID-19 still in the acute phase of the disease, and using the Brief Memory and Executive Test, revealed 81% patients with cognitive impairment^19^. A follow-up study with 135 patients previously hospitalized with COVID-19, revealed that, after three months of infection, patients still exhibited cognitive impairment, assessed using the Montreal Cognitive Assessment (MoCA), which varied according to the clinical severity of the disease, reaching 29% in the more severe cases^20^. Additionally, a study that evaluated 50 outpatients four months after infection demonstrated a worse performance in attention and working memory tests, when compared to 50 participants who had not been infected^21^.

The clinical evaluation of these patients should preferably include a detailed neuropsychological evaluation with tests validated for the population in question, taking into account their education^8^ ^,^ ^16^. The importance of extensive neuropsychological batteries is well exemplified by studies that have demonstrated the insensitivity of cognitive screening tests^22^. García-Sánchez et al. evaluated 63 patients, including 33 previously hospitalized patients, six months after infection and reported attention as the most affected cognitive domain from an extensive neuropsychological assessment^8^. Most studies to date are cross-sectional, making it difficult to determine the duration of these symptoms, but a longitudinal study by Del Bruto et al. demonstrated resolution of cognitive impairment in long COVID patients after one year^7^. Still, in this sense of longitudinal studies, Ballouz et al. evaluated the self-reported symptoms of 1,106 patients who had COVID-19 and compared them to 628 patients without infection at 6, 12, 18 and 24 months post-infection^23^. In this study, the authors found the persistence of several symptoms, including impaired attention and memory in 17.2% of participants even after 24 months^23^. The persistence of cognitive impairment in approximately half and a third of 226 previously hospitalized patients, 3 and 12 months after infection, respectively, was also reported^24^.

The pathophysiology responsible for these symptoms may involve endothelial injury, ischemic brain changes, neuroinflammation, or direct viral invasion^6^ ^,^ ^25^ ^,^ ^26^ ^,^ ^27^. Thus, the search for possible biomarkers that signal a pathophysiology underlying this symptomatology involves tools such as neuroimaging exams and fluid analysis^28^ ^,^ ^29^. With regard to neuroimaging studies in these patients, structural and functional alterations have been described in patients with this condition^28^ ^,^ ^29^ ^,^ ^30^. In a case study with severe cases of COVID-19, patients’ neuroimaging tests showed microbleeds in deep gray matter, cerebellum, and corpus callosum, and extensive white matter changes were also described, suggestive of possible COVID-19 encephalopathy^31^. Douaud et al. described, using brain MRI, gray matter atrophy in the orbitofrontal cortex and parahippocampal gyrus in 401 post-COVID-19 patients compared with pre-COVID-19 examination of the same patients^28^. Other promising magnetic resonance imaging (MRI) techniques in the study of these patients are diffusion tensor imaging (DTI) and arterial spin labeling, which were even used in patients with long COVID by Kim et al. and Teller et al., respectively^32^ ^,^ ^33^. In the first study, low diffusion restriction in frontal circuits and high diffusion restriction in cerebellum were found in patients with COVID-19 compared to controls^32^. In the second study, Tellet et al. demonstrated reduced cerebral blood flow (CBF) in the thalamus, basal ganglia, and orbitofrontal cortex in COVID-19 patients compared to controls^33^. With regard to possible fluid biomarkers, in patients with long COVID there is an activation of the inflammasome mediated by SARS-Cov-2 and a subsequent dysregulated inflammatory response, with consequent persistent elevation of pro-inflammatory cytokines such as IL-1, IL-6 and TNF- alpha^26^ ^,^ ^34^ ^,^ ^35^ ^,^ ^36^. Furthermore, astrocytic activation biomarkers and neurodegeneration markers may also be elevated in patients with neuro long COVID^37^. Finally, research on specific Alzheimer’s disease biomarkers is also relevant, mainly to establish the risk of developing future neurodegenerative diseases^38^.

## SLEEP DISORDERS

Sleep disorders have been reported in up to 26% of patients in long COVID cohorts, particularly insomnia^39^. A possible explanation for persistent sleep disorders after COVID-19 might be related to prolonged dysfunction of brainstem nuclei^40^. This dysfunction could be explained by a high concentration of ACE2 receptors (which are used by SARS-CoV-2 to enter cells) in the brainstem^40^. Some of these brainstem nuclei are involved with sleep-wakefulness regulation^40^.

Sleep disturbances experienced during the COVID-19 pandemic vary from insomnia to hypersomnia, nightmares and even the worsening of sleep-disordered breathing, such as obstructive apnea^41^. The impact of sleep was observed worldwide whether due to the direct effect of the infection or the change in the circadian cycle imposed by the new routine started in quarantine^41^.

Chronic insomnia was the most common sleep disorder after COVID infection, in line with other studies of long-COVID patients^42^. Social isolation, stress, anxiety, depression, persistent inflammatory response, and corticosteroid use might justify the high prevalence in long COVID. It is possible that changes associated with the acute infection might have precipitated dysfunctional habits, like excessive caffeine consumption or bad sleep hygiene.

As potential neuroimmunological diseases, hypersomnia (narcolepsy, Kleine-Levin syndrome [KLS] and idiopathic hypersomnia [HI] can be triggered by external factors, such as upper airway infection, as occurred with H1N1 influenza or neuroimmunological response to vaccination^40^ ^,^ ^43^ ^,^ ^44^.

## NEUROPSYCHIATRIC SYMPTOMS

Neuropsychiatric symptoms including delirium, mood swings and psychosis have been exacerbated due to COVID-19^45^. It is observed that the acute respiratory syndrome and associated relative hypoxia result in the worsening of attention, executive function, and verbal memory^46^. It is evident that not only individuals infected with SARS-COV-2 but also family members and the healthy population, since the beginning of the pandemic in Wuhan in 2020, have had psychiatric impairments due to the situation prevailing in the period, mainly related to behavioral changes of social and sanitary restriction^45^ ^,^ ^47^. In the same manner, long COVID can also promote feelings of isolation and trauma in an individual and other nonpsychiatric symptoms can trigger psychological burdens, such as fatigue leading to reduced motivation^48^. The aetiology of the neuropsychiatric causes of COVID-19 in regard to the interaction of the virus with the central nervous system is diverse and includes imbalanced neurotransmitters, disruption of the blood-brain barrier, promotion of hypoxia and unbalanced immune response. Furthermore, it represents a new context of a virus of global impact on society^45^ ^,^ ^46^. It’s important to highlight that although the biological mechanisms that alter brain structure and function are important in augmenting neuropsychological changes, minor changes in non-eloquent regions of the brain can also promote subtle yet complex effects^48^. Depressive symptoms, in varying degrees, and clinically relevant post-traumatic stress disorder (PTSD) have been documented in patients who had COVID-19 even 22 months after infection, and a correlational analysis showed that stronger PTSD symptoms were correlated with poor performance in Weigl’s test and attentional matrices^49^. This study also showed that psychological well-being and a perceived better quality of life led to better verbal learning and executive functions, respectively^49^.

A cohort of 701 adult patients who recovered from moderate to severe COVID-19 revealed that 7 to 11 months after hospital discharge common mental disorders were prevalent (30%), generalized anxiety disorder (15.1%), mixed depressive and anxiety disorder (13.5%), depression (7.5%), obsessive-compulsive disorder (3.1%), specific phobia (2.1%), social phobia (0.8%) and panic disorder (0.8%). The psychiatric assessment also reported a prevalence of PTSD (13.4%), last-year suicidal attempt (2.4%), and last four weeks of suicidal ideation (10.1%). Interestingly they found that patients with ageusia and anosmia displayed more psychiatric and cognitive impairment. However, although chemosensory deficits were statistically significantly associated with poor cognitive function, for psychiatric disorders it was not the case^47^.

Busatto et al. assessed 579 adults who had been hospitalized with COVID-19, for post-acute sequelae of SARS-CoV2 (PASC) 6 to 11 months after infection. They found that alongside fatigue, psychiatric and cognitive manifestations were the most frequent symptoms, and this was irrespective of comorbidities. Symptoms of post-traumatic stress, depression, memory loss, anxiety, lack of concentration, and insomnia were the prominent central nervous system (CNS) manifestations significantly associated with PASC. Furthermore, high levels of PCR and D-dimer were equally and significantly correlated with PASC symptoms^50^. The elevated levels of PCR and D-dimer described support the hypothesis that dysregulated inflammatory and immune pathways play an important role in the pathophysiology of PASC with persistent systemic inflammation promoting extended physical, psychiatric, and cognitive debilitated manifestations through pro-inflammatory agents infiltrating the CNS^51^ ^,^ ^52^. This is facilitated by organs that possess incomplete blood-brain barrier or abnormally permeable portions due to cytokine damage^53^. The persistence of the genetic material of the virus is also implicated in decreased excitability of the hippocampus and neuronal losses in memory processing areas^54^ ^,^ ^55^. Within this scenario, it is reported that the presence of genes linked to the immune response may contribute to a greater predisposition in the development of neurological sequelae, among them neurodegenerative diseases^56^.

In addition, cytokines also play an essential role in the weakening of the blood-brain barrier. IL-6 is probably one of the leading causes of such a process, since it was found that its activity induces a decrease in the expression of intra-endothelial adhesion proteins of the brain microvasculature in vitro, increasing paracellular permeability. It is also important to note that anti-IL-6 is found during in-vivo ischemia, suggesting that IL-6 may be responsible for the increased permeability of the blood-brain barrier after injury. Finally, some studies reported increased IL-6 in cerebrospinal fluid of patients with neurological impairments from COVID-19 compared to control individuals^57^.

As for neuropsychiatric manifestations, the recurrence of insomnia, psychosis, and mood swings are highlighted. Given the “cytokine storm” caused by SARS-CoV-2, patients may present encephalopathy, as well as rare cases of encephalitis, causing neuropsychiatric changes. Individuals with previous psychiatric comorbidity, sequelae such as anxiety, depression, post-traumatic disorder, and insomnia are more frequently described, and associated with worse scores on psychopathological scales^46^ ^,^ ^58^. In addition, these findings point to an important correlation between cognitive function and psychiatric outcomes, where one can influence the other.

## OLFACTORY DYSFUNCTION

Loss of smell is one of the most common symptoms of COVID-19, with prevalence ranging of 11-84% in the acute phase of the disease^59^. Hyposmia can occur during the acute phase of the disease or after 12 weeks of the initial condition^3^ ^,^ ^59^. A systematic review selected studies that evaluated the recovery of anosmia in patients after 1, 2, and between 3-6 months after COVID-19, showing hyposmia in 37.4, 36.7, and 36.5% of patients, respectively^60^. The pathophysiology involved in this symptomatology involves blockage of transit of odorants to the olfactory receptors from nasal congestion and olfactory bulb injury from cytokines^61^. Table 1 summarizes these and the previous sections’ common symptoms.

**Table 1.** Symptoms summary.

Topic	Symptoms
Cognitive symptoms	Attention, memory, executive functions and processing speed complaints in long COVID phase^3^. Akinetic mutism associated with frontal hypometabolism in the brain in acute phase was also described^18^.
Sleep disorders	Insomnia, hypersomnia, nightmares, worsening of sleep-disordered breathing, change in the circadian cycle^41^ ^,^ ^44^.
Neuropsychiatric symptoms	Exacerbation of delirium, mood swings, psychosis in acute phase. Behavioral changes of social and sanitary restriction^46^. Fatigue with decreased motivation^51^. Depressive symptoms, post-traumatic stress disorder (PTSD), generalized anxiety disorder^49^.
Olfatory disfunction	Hyposmia and anosmia^59^ ^,^ ^60^.

## LIMITATIONS

Several neurological symptoms are present in patients with long-term COVID-19, some of which are not described in this review, such as headache, cerebrovascular diseases, and autoimmune and neuromuscular diseases.

In conclusion, long COVID can present with various neurological and psychiatric symptoms, including cognitive decline, sleep disorders, olfactory dysfunction, anxiety, and depression. Understanding at what stage these manifestations occur and their consequences will assist healthcare professionals in specialized centers to provide better diagnostic and therapeutic guidance. Furthermore, it is important to highlight that an understanding of how these manifestations are triggered by viral infections is essential to assist patients who are predisposed or already harbor some of these cognitive and/or psychiatric conditions prior to infection. The interplay between biological, physical, cognitive, and psychiatric factors is also essential once these have been observed in combination. The use of more neuroimaging and genetic techniques alongside the existing protocols to determine a causative effect can assist in the early detection of signs and symptoms of long COVID.
